# Patient-reported outcomes and medical device evaluation: from conception to implementation

**DOI:** 10.1186/s41687-022-00463-w

**Published:** 2022-05-24

**Authors:** Christina M. Webber, Brittany Caldwell, Fraser D. Bocell, Anindita Saha, Michelle E. Tarver

**Affiliations:** Center for Devices and Radiological Health, U.S. Food and Drug Administration, Silver Spring, MD USA

**Keywords:** Patient-reported outcome, Medical device, Evaluation, Safety, Effectiveness, FDA

## Abstract

**Background:**

The insights gleaned from patient-reported outcomes (PROs) have implications across the healthcare ecosystem, from clinical investigations to evaluate the safety and effectiveness of medical devices to clinical care and reimbursement decisions. The U.S. Food and Drug Administration’s (FDA) Center for Devices and Radiological Health (CDRH) hosted a public meeting in September 2020 discussing how PROs can be used in medical device evaluation throughout the total product life cycle, as well as methods for developing and modifying PRO instruments to ensure they are fit-for-purpose. This commentary presents key points of discussion from the meeting, providing insight into the increased interest in PRO data to support medical product development while also exploring future opportunities of incorporating PRO data throughout healthcare.

**Main Body.:**

Thoughtful use of fit-for-purpose PRO instruments to integrate the patient’s voice into clinical care paradigms, medical device development, regulatory decisions, and reimbursement and coverage decisions were emphasized throughout the meeting. Existing PRO instruments may be used if the context of use is appropriate. Modifications to an existing PRO instrument may also be explored to ensure the instrument is fit-for-purpose in a new context of use. Development of a novel PRO instrument may be necessary to capture attributes in a new patient population or application. Multi-stakeholder collaborations, of which patients are a key component, create efficiencies in the development and modification of PRO instruments.

**Conclusion:**

Continued multi-stakeholder collaborations bringing together researchers, clinicians, patients, regulators, and payers are critical to further advance the inclusion of the patient voice incorporating PRO instruments throughout the healthcare ecosystem in an efficient manner that is least burdensome to patients.

## Background

Patients offer a unique and valuable perspective on how their conditions are impacted by medical devices. Pain and fatigue are both examples of concepts for which only patients can accurately report. A patient-reported outcome (PRO) is a measurement “based on a report that comes directly from the patient about the status of the patient’s health condition without amendment or interpretation of the patient’s response by a clinician or anyone else” [1]. PRO instruments may be in the form of rating scales, questionnaires, and diaries. These outcomes can be used as stand-alone data or they may complement other outcome measures, helping to form a more well-rounded picture of the quality of life including the health status of patients.

The unique perspective provided by PRO data has applications throughout the total product life cycle with impacts across the healthcare ecosystem from the premarket evaluation of devices through reimbursement and use of a device in clinical care. PRO instruments may be used in clinical investigations conducted by medical device manufacturers to help evaluate the safety and effectiveness of higher risk medical devices, typically Class II or III devices (as defined in 21 CFR 860.3.c) providing evidence for pre-market benefit-risk determinations or post-market surveillance and signal monitoring efforts. The clinical setting also may benefit from including PRO instruments to help inform early diagnosis and preventive care, assist with shared decision-making, or evaluate treatments. Information from PRO instruments can also be used to inform reimbursement and coverage decisions by payers and in health technology assessments.

In September 2020, the U.S. Food and Drug Administration’s (FDA) Center for Devices and Radiological Health (CDRH) hosted a public meeting, *Patient-Reported Outcomes and Medical Device Evaluation: From Conception to Implementation*, bringing together regulators, members of the medical device industry, academics, patients, providers, and other key stakeholders to discuss how PRO data can be used throughout the total product life cycle of medical devices, as well as methods for developing and modifying PRO instruments to ensure they are fit-for-purpose [2]. This commentary presents key points of discussion from the meeting, providing insight into the increased interest in PRO data observed in medical product development while also exploring future opportunities for using PRO data throughout the healthcare ecosystem.

## Main text

### Importance of PRO assessment throughout the healthcare ecosystem

Assessment of PROs can measure areas that are important to patients, adding rich context to regulatory, clinical, and payer decisions. Data from PRO instruments can be stand-alone or complementary to other clinical outcomes when they are reliable and robust in a specific context of use. The integration of PRO assessments throughout the entire healthcare ecosystem, from medical device evaluation through clinical care to reimbursement and coverage decision-making, was one focus of the meeting.

#### Regulatory

Data from PRO instruments can be used to aid in the evaluation of the safety and effectiveness of medical devices as either stand-alone or complementary evidence, with applications across the total product life cycle from premarket evaluation through post-market surveillance and/or label expansion. A series of legislative and regulatory activities, including CDRH’s guidance on selecting, developing, modifying, and adapting PRO instruments as well as the 21st Century Cures Act and the resulting patient-focused drug development guidance series, collectively are ushering scientifically valid patient experience data into medical product evaluation [3, 4]. CDRH encourages the voluntary inclusion of fit-for-purpose PRO instruments in clinical investigations of medical devices to support the evaluation of both safety and effectiveness [3, 5]. When a PRO instrument is considered fit-for-purpose, its validation, or the evidence to support the interpretation of the score, is sufficient to support its use in a specific context [1]. While a clinical care team can evaluate a patient to determine the observable aspects of a patient’s health status, only a patient can report on their experience with symptoms, such as pain intensity and anxiousness.

A recent CDRH analysis found that, on average, half of the devices receiving premarket approvals between October 1, 2014 and September 30, 2019 contained data from at least one PRO instrument [6]. CDRH has outlined approaches to encourage the development and use of “relevant, reliable, and sufficiently robust PRO instruments using a least burdensome approach” in a guidance document [3]. Unlike drugs, medical devices are subject to the least burdensome provision which means the premarket evaluation is done in a manner that eliminates unnecessary burdens while maintaining the statutory requirements for clearance and approval [7]. Further embodying the least burdensome principle, if sufficient validation evidence exists for a given context of use, an existing PRO instrument may be leveraged in lieu of developing a new instrument. If a novel PRO instrument is needed, then efficient development approaches are encouraged, including multi-stakeholder collaborations and harnessing real-world data platforms (for example, patient-driven registries). Early and frequent engagement with FDA through the pre-submission program, offers researchers and industry an opportunity to present and discuss proposed approaches to PRO instrument development and evaluation.

#### Clinical care

In addition to industry’s use of PRO instruments to support the safety and effectiveness of a medical device in a regulatory submission, PRO instruments can be impactful to assess real-world performance of a medical device after receiving marketing authorization and used in clinical care. This was particularly evident with the rise in telehealth during the coronavirus disease 2019 (COVID-19) pandemic where PRO instruments provided an opportunity for patients to remain connected with their providers. The data from PRO instruments can supplement common clinical measurements and tests like blood pressure, joint range of motion, and electroencephalography. It provides information beyond what is gleaned from those tests to create a more comprehensive description of a patient’s health status. A longitudinal look at a patient’s health status is possible if PRO instruments are delivered through an electronic format, such as a website or mobile application, and integrated as part of a carefully designed and implemented monitoring plan. This outside-the-office perspective can provide real-time information on a patient’s health status, as well as help serve as a flag for when a patient might need additional care or be used as a discussion tool in shared decision-making.

#### Coverage and reimbursement

Payers and health technology assessment bodies may consider PRO data as part of the totality of evidence considered for coverage decisions when evaluating the effect of a medical device or the overall treatment of a disease or condition. They may look to peer-reviewed literature supporting a given PRO instrument as well as other data sources presented as evidence to help frame their evaluation [8]. Reimbursement and coverage decisions may rely, in part, on PRO assessments to describe the impact of a treatment on clinical outcomes and quality of life.

### Fit-for-purpose PRO instruments

With the range of possible uses for PRO instruments, the need to demonstrate a PRO assessment is fit-for-purpose in different situations was consistently discussed. Scientifically valid evidence can demonstrate that a PRO instrument is fit-for-purpose in a given context of use. This holds true when developing a novel PRO instrument, as well as when modifying or adapting an existing instrument. In addition to the psychometric properties (e.g., validity, reliability) of the PRO instrument, consideration should be given to the application of the instrument, its intended use, handling of item and scale level missing data, and how the PRO instrument can be integrated into existing clinical care paradigms and electronic health records. Panelists throughout the meeting discussed the importance of revisiting the evidence supporting PRO instruments to confirm existing validity evidence, including evidence of content validity, as clinical practice and standards of care advance with the introduction of technological innovations, novel medical products, and evolving methodologies. The patient experience is continuously evolving with these advances.

Along with changes in patient experience due to these advancements, the continued application of PRO data in the regulatory, clinical, and reimbursement spaces may necessitate continued collection of validity evidence, including evidence of content validity, for the instruments in different contexts of use. Experts at the meeting noted that while using portions of instruments with existing validity evidence could improve the efficiency of PRO instrument development or use, further exploration is warranted to determine the effect this change may have on the interpretability and/or utility of an instrument in a given context of use. Existing evidence should be evaluated for its applicability to specific portions of the existing instrument and whether additional evidence is needed.

### Modifying an established PRO instrument

In addition to using portions of existing instruments, modification may be an efficient method for ensuring an instrument is fit-for-purpose. Modifications to an existing PRO instrument can broaden its applicability to a different population or technology. When a PRO instrument is intended to be used in a regulatory context, CDRH encourages the modification and development process to occur in a least burdensome manner for stakeholders involved in development (e.g., medical device sponsors, PRO instrument developers, patients), leveraging precompetitive collaborations where appropriate [3].

Minor modifications to existing PRO instruments could support their use in other populations or subgroups, keeping in mind that the amount and types of changes will impact the validity evidence needed to support use of the modified instrument. Demographic considerations are particularly important, especially since lived experiences or symptoms experienced with a disease or condition may differ for various groups. Gender, race, ethnicity, socioeconomic status, developmental status, age, time spent living with a disease or condition, and health literacy are some important factors mentioned during the discussion as useful when assessing the appropriateness of a PRO instrument in a specific context of use.

One example focused on modifying an established PRO instrument, the Kansas City Cardiomyopathy Questionnaire (KCCQ), for use in a population with an 8^th^-grade reading level, the level recommended for PRO instruments [9]. While panelists discussed potential modifications within the context of the KCCQ, the conversation could be extended to considerations when modifying other PRO instruments. The panel of experts noted that when modifying an existing PRO instrument, it is important to consider the existing body of evidence and how the modifications impact the overall use and interpretation of the instrument and scores. Also emphasized by the panel was the need to consider how the results of a new or modified PRO instrument can be communicated not only to clinicians, but also to patients.

Furthermore, the panel pointed out clinically meaningful differences in a PRO instrument’s scores should be explored during development or modification of an existing PRO instrument, as part of the evidence to support the interpretation of the score. Additionally, panelists highlighted that patients should be consulted throughout the process to ensure that the instrument measures attributes they find important. Patient consultation can also help developers use accessible and understandable terminology. The panel noted that while modifications to an existing PRO instrument may be appropriate for some situations, it may not be appropriate in all. For example, pediatric patients and adult patients can experience different symptoms of heart failure. Modifications to an existing instrument assessing heart failure symptoms in adults may not fully capture the impact the condition has on a pediatric population, so development of a new PRO instrument may be warranted.

### Developing a novel PRO instrument

If an existing PRO instrument or modification does not sufficiently meet the requirements of a desired application or the need for modification is too great, then development of a new instrument could be considered. Three case studies covering the development of new PRO instruments were discussed during the meeting, bringing together the instrument developers and regulators from the relevant product areas. Each explained their considerations during development, including the motivation and purpose for developing the new PRO instrument. All instruments were developed by a multidisciplinary team, including psychometricians, qualitative methods experts, clinicians, other quality of life researchers, and patients.

Development of novel PRO instruments were spurred by clinician needs, patient demands, and inadequate assessment of the treatment context. For example, the BREAST-Q and other Q-portfolio instruments were developed because clinicians noted a lack of instruments designed to measure outcomes of patients treated by plastic surgeons, including those undergoing aesthetic procedures, breast surgery, or weight loss surgery [10]. The instruments can help initiate and facilitate conversations with patients about topics that may be difficult to speak freely about like satisfaction with appearance, body image, social functioning, and sexuality. In another example, patient reports of visual symptoms following laser-assisted in situ keratomileusis (LASIK) during a federal advisory committee meeting prompted the FDA to collaboratively develop a new PRO instrument, the Patient-Reported Outcomes With LASIK (PROWL) questionnaire [11]. The last new PRO instrument discussed at the meeting, the Insulin Dosing Systems: Perceptions, Ideas, Reflections, and Expectations (INSPIRE) questionnaire, was developed to assess the psychosocial outcomes associated with automated insulin dosing, a gap observed in clinical investigations for the medical devices [12]. Each of these PRO instruments were designed to fill a measurement gap that was important in understanding treatment impacts.

### Multi-stakeholder collaboration

The importance of multi-stakeholder collaboration and engagement throughout the development and/or implementation of a PRO instrument was stressed by experts during the meeting. Active stakeholder engagement in both the development and use of a PRO instrument can help build confidence in the data supporting the PRO instrument and therefore trust in the PRO instrument, regardless of the application space. The experts recommended bringing together stakeholders like medical device manufacturers, researchers, clinicians, patients, regulators, payers, and others during development. The PRO instrument development process is iterative and requires flexibility and time from all stakeholders involved to develop a relevant PRO instrument. Comprehensive stakeholder engagement from the beginning of PRO instrument development or modification aids in aligning the group’s priorities and expected outcomes. While each stakeholder may come to the table with their individual needs and priorities, all parties can unite to pursue their common goal of developing a PRO instrument that can be used to help assess patients’ quality of life and functioning. Experts noted that an explicitly defined common goal also aids in balancing stakeholders’ individual needs with the finished instrument’s appropriateness for a given context of use.

Standard terminology may differ between stakeholders, so having collaborative discussions with all parties at the table could improve interpretability and understanding of the instrument and its results. Since healthcare providers and patients may be unfamiliar with PRO assessment, panelists recognized that additional efforts may be necessary to improve the instrument’s use, especially if the instrument is to be used to assist with shared decision-making. While precompetitive collaborations require long-term planning and commitment by multiple parties, they offer the opportunity to distribute resource requirements across the group, thereby potentially reducing an individual party’s burden.

The importance of focusing specifically on patient engagement and ways that patients can be involved during development was further explored by the panel. First, patients can join other stakeholders as collaborators, contributing to the development of a PRO instrument as an equal partner with other stakeholders. Second, patients can volunteer as participants during the content generation phase of development. Patients are the experts in what it is like to live with a particular disease or condition, so patient engagement throughout instrument development is crucial to providing evidence of content validity and applicability of the instrument. Experts suggested those interested in developing or modifying PRO instruments reach out to relevant patient advocacy groups, clinicians, and community organizations like churches or community centers to connect with patients interested in being part of the development process. Including patients that reflect the diversity of the real-world patient population bolsters the relevance of the resulting instrument. Development, modification, and adoption of PRO instruments throughout the healthcare ecosystem can be realized in an efficient manner through precompetitive collaboration while minimizing burden on patients. Collaboration will be essential for future technological advances in PRO instrument delivery and integration of PRO data into electronic health records.

## Conclusions

Throughout the meeting, panelists emphasized the importance of thoughtful use of fit-for-purpose PRO instruments to integrate the patient’s voice into clinical care paradigms, medical device development, regulatory decisions, and reimbursement and coverage decisions. Depending on the context of use, an existing PRO instrument may be appropriate. Thoughtful modifications to an existing PRO instrument may also be explored to ensure the instrument is fit-for-purpose in a new context of use. Development of a novel PRO instrument may be necessary to fill the measurement gap, particularly in emerging technological areas that prompt new concepts or where well-defined instruments are not available to measure existing concepts. No matter the approach to ensuring a fit-for-purpose instrument, continued multi-stakeholder collaborations bringing together researchers, clinicians, patients, regulators, and payers are critical to further advance the inclusion of the patient voice using PRO assessment throughout the healthcare ecosystem in a least burdensome and efficient manner. The patient experience is not static, nor should the PRO instruments used throughout the healthcare ecosystem to measure their experience be static. Patients are a key component of multi-stakeholder collaborations that can be used to realize efficiencies in the development and modification of PRO instruments. All stakeholders working together in a flexible manner can help ensure PRO instruments are fit-for-purpose in their use and interpretation.

## Data Availability

Not applicable.

## Abbreviations

PRO: Patient-reported outcome
FDA: U.S. Food & Drug Administration
CDRH: Center for Devices and Radiological Health
COVID-19: Coronavirus disease 2019
KCCQ: Kansas City Cardiomyopathy Questionnaire
LASIK: Laser-assisted in situ keratomileusis
PROWL: Patient-Reported Outcomes with LASIK
INSPIRE: Insulin Dosing Systems: Perceptions, Ideas, Reflections, and Expectations
